# LDHA-lactate axis modulates mitophagy inhibiting CSFV replication

**DOI:** 10.1128/jvi.00268-25

**Published:** 2025-04-23

**Authors:** Sen Zeng, Zipeng Luo, Wenhui Zhu, Zhanhui Zhang, Ruibo Zhao, Shuaiqi Zhu, Qi Qiu, Nan Cao, Xinliang Fu, Wenjun Liu, Shuangqi Fan, Cheng Fu

**Affiliations:** 1 College of Animal Science & Technology, Zhongkai University of Agriculture and Engineering 554665, Guangzhou, China; 2 College of Veterinary Medicine, South China Agricultural University 547855, Guangzhou, China; University of Michigan Medical School, Ann Arbor, Michigan, USA

**Keywords:** LDHA, lactate, mitophagy, JAK-STAT, CSFV

## Abstract

Lactate dehydrogenase A (LDHA) plays a crucial role in regulating lactate synthesis in various biological processes. Lactate, a byproduct of glycometabolism, has been recognized as a unique molecule with implications in both metabolism and immunity. Classical swine fever (CSF), caused by the classical swine fever virus (CSFV), is a highly contagious and severe infectious disease that primarily affects pigs. Prior research has shown that CSFV infection disrupts the normal glycolytic process, leading to an accumulation of lactate within the host. Nevertheless, it remains unclear whether there is mutual regulation between the CSFV and LDHA-lactate axis. Here, we have found that CSFV infection increases LDHA expression *in vivo* and *in vitro*, which may be attributed to attenuated ISGylation of LDHA. Furthermore, CSFV infection induces L-lactate production via LDHA dependence *in vitro*. The cellular biology research on LDHA has revealed that LDHA not only localizes to the mitochondria but also inhibits PINK1-Parkin-mediated mitophagy. Through various experimental techniques such as western blot to detect mitophagy marker proteins, laser confocal microscopy to observe the flow of mitophagy, and transmission electron microscopy to assess changes in the number of mitochondria enclosed within autophagosome-like vesicles, it has been discovered that the addition of exogenous lactate can inhibit PINK1-Parkin-mediated mitophagy. Importantly, we have observed that lactate activates the JAK1-STAT1-ISG15 network and suppresses CSFV replication by antagonizing CCCP-induced mitophagy. These results represent the first report on the mechanisms through which the LDHA-lactate axis regulates mitophagy, the JAK-STAT pathway, and CSFV replication. This study provides novel insights into the roles of the LDHA-lactate axis in glycometabolism and viral replication.

**IMPORTANCE:**

This research unveils how CSFV interacts with cellular metabolism through LDHA. By revealing LDHA’s dual role and how lactate influences cellular processes during CSFV infection, this study uncovers new pathways for viral replication. These findings not only deepen our understanding of viral-host interactions but also open doors for innovative antiviral strategies centered around manipulating cellular metabolism.

## INTRODUCTION

Lactate, a well-known metabolic product of glycolysis, is primarily catalyzed by lactate dehydrogenase A (LDHA). Emerging studies have demonstrated that LDHA and lactate play a vital role in viral infection (1, 2). Elevated LDHA and lactate have been observed in severe acute respiratory syndrome coronavirus 2 (SARS-CoV-2) and influenza avian virus (AIV) infections (2–4). Lactate directly binds the transmembrane domain of the mitochondrial antiviral signaling protein (MAVS) and blocks the production of type I interferon (IFN-I) in vesicular stomatitis virus and Sendai virus infection. In addition, LDHA has been identified as an inhibitor of the retinoic acid-induced gene I (RIG-I)-like receptor (RLR) signaling pathway, which consequently hinders the localization of MAVS to the mitochondria (5). These studies confirm that lactate is a natural immunomodulatory metabolite.

Classical swine fever virus (CSFV) is a single-stranded positive-stranded RNA virus, belonging to the genus *Pestivirus* within the family *Flaviviridae* (6, 7). The genome of CSFV encodes a viral polyprotein which can be cleaved to generate four structural proteins (E^rns^, E1, E2, and C) and eight non-structural proteins (N^pro^, p7, NS2, NS3, NS4A, NS4B, NS5A, and NS5B) (8–10). Previous reports of proteomic analyses have indicated that CSFV infection in PK-15 cells improved the glycolytic process by increasing phosphoglycerate mutase 1 (PGAM1), glyceraldehyde-3-phosphate dehydrogenase (GAPDH), and triosephosphate isomerase (11). Although the pathogenesis mechanisms of CSFV have been conducted, the precise pathways largely remain to be elucidated.

Mitophagy is the process in which mitochondria are selectively degraded by autolysosomes, which has both positive and negative effects on the pathogenesis of viral diseases (12). Our previous data have shown that CSFV infection modulates mitophagy and causes lactate accumulation via lactate dehydrogenase B (LDHB) (13). Moreover, CSFV activates mitophagy through PTEN-induced kinase 1 (PINK1)-Parkin and mitochondrial dynamics pathway, which, in turn, regulates inflammatory cytokine production and CSFV-sustained replication (13).

The Janus kinase (JAK)-signal transducer and activator of transcription (STAT) pathway plays a central role in viral infection. Following virus detection and interferons (IFNs) production, IFNs bind with IFN receptor 1/2 and activate JAK and tyrosine kinase 2 (TYK2), leading to recruitment and phosphorylation of STAT1/2 and promoting the transcription of interferon-stimulated genes (ISGs) (14). Furthermore, studies have found that upon sensing CSFV infection, the ubiquitin-proteasome system mediates STAT1 protein degradation, leading to inhibition of the JAK-STAT pathway and ISG transcription (15, 16). It is noteworthy that there is a mutual regulation between mitophagy and the JAK-STAT pathway (17). A recent study found that IFNs play a bridge role between the modulation of autophagy and the JAK-STAT pathway (18). Moreover, autophagy facilitates IFN-γ-induced JAK2-STAT1 activation and inflammation (19). Therefore, extensive regulation between mitophagy and the JAK-STAT pathway may play a vital role in viral infection.

Here, we discovered for the first time that CSFV infection causes a significant increase in LDHA *in vivo* and *in vitro*. Molecular biology analyses suggested that the LDHA-lactate axis inhibits the PINK1-Parkin-mitophagy network and activates the JAK1-STAT1-ISG15 network, which in turn inhibits CSFV replication. These results will provide new insights for elucidating the mechanisms of the LDHA axis on metabolism and viral replication.

## MATERIALS AND METHODS

### Cell culture

The swine kidney cell line PK-15 (ATCC, CCL-33) cells were cultured in Dulbecco’s modified Eagle’s medium (Thermo Fisher Scientific, C11995500BT) supplemented with 10% fetal bovine serum (FBS) (Thermo Fisher Scientiffc, 10099141). The porcine alveolar macrophages 3D4/2 (ATCC, CRL-2845) were maintained in complete RPMI 1640 medium (Thermo Fisher Scientific, C11875500BT) containing 10% FBS. All cells were cultured at 37°C in a 5% CO_2_ incubator.

### Reagents and antibodies

The chemical reagents used in this study are as follows: dimethyl sulfoxide (DMSO; Sigma-Aldrich, V900090), carbonyl cyanide 3-chlorophenylhydrazone (CCCP; Sigma-Aldrich, C2759) and L-(+)-Lactic acid (Sigma-Aldrich, L1750).

The primary antibodies used in this study are as follows: rabbit polyclonal anti-MAP1LC3B (Cell Signaling Technology, 2775), VDAC1 rabbit monoclonal antibody (Beyotime, AF1027), TOMM20 rabbit monoclonal antibody (Beyotime, AF1717), mouse monoclonal anti-CSFV E2 (Jai Balajee Trading Co., 9011), rabbit anti-LDHA antibody (Abmart, T58276), α-Tubulin mouse monoclonal antibody (Beyotime, AF2827), mouse monoclonal anti-Flag (Beyotime, AF519), mouse monoclonal anti-HA (Beyotime, AH158), rabbit monoclonal anti-Flag (Cell Signaling Technology, 14793), rabbit monoclonal anti-HA (Cell Signaling Technology, 3724), Stat-1 α/β rabbit polyclonal antibody (Beyotime, AF0288), phospho-STAT1 (Tyr701) rabbit monoclonal antibody (Abmart TA3300), phospho-Jak1 (Tyr1022/1023) rabbit polyclonal antibody (Beyotime, AF5857), ISG15 rabbit polyclonal antibody (KleanAB, P111424), rabbit monoclonal anti-PINK (Cell Signaling Technology, 6946T), mouse monoclonal anti-Parkin (Cell Signaling Technology, 4211S), and rabbit monoclonal anti-p62 (Cell Signaling Technology, 39749).

Secondary antibodies used for immunofluorescence were Cy3 goat anti-rabbit IgG (Beyotime, A0516), Cy3 goat anti-mouse IgG (Beyotime, A0521), FITC goat anti-rabbit IgG (Beyotime, A0562), and FITC goat anti-mouse IgG (Beyotime, A0568). Secondary antibodies used for western blot analysis were HRP-conjugated goat anti-mouse IgG (Beyotime, A0216) and HRP-conjugated goat anti-rabbit IgG (Beyotime, A0208). Mouse polyclonal anti-CSFV N^pro^ was provided by Sen Zeng, a doctoral student in our lab.

### Virus infection and viral titer detection

The CSFV strain (Shimen) used in this study was isolated from pigs with typical symptoms by our laboratory and propagated in PK-15 cells. PK-15 cells were cultured to around 80% confluence in cell culture plates and then infected with CSFV at an MOI of 1.0. After 2 h, the inoculum was removed, cells were washed with sterile phosphate-buffered saline (PBS, Solarbio, P1022), and incubated in fresh medium at 37°C until harvest. Viral titers were calculated using the Kaerber method and expressed as 50% tissue culture infectious doses (TCID_50_) per milliliter based on indirect immunofluorescence (IFA) (20).

### Animal experiments

The animal infection assay was performed as previously described by Zhu et al. (21). All operations were carried out in accordance with the regulations of the Laboratory Animal Ethics Committee of South China Agricultural University. Briefly, a total of six 2-month-old piglets, free of porcine reproductive and respiratory syndrome, pseudorabies virus, and porcine parvovirus infection, were challenged with 10^5^ TCID_50_ of CSFV Shimen strain. Piglets are raised with positive-pressure filtered air in a specific pathogen-free animal facility. Before and after CSFV infection, anterior vena cava blood in piglets was aseptically collected into heparin sodium anticoagulant tubes every other day. Animal experiments are mainly used for immunohistochemistry (IHC) here.

### Immunohistochemistry

The experimental pigs were sacrificed at 7 dpi of CSFV infection for IHC. A macroscopic examination was performed immediately after the sacrifice of the experimental pigs. Macroscopic photographs of the indicated immune organs and non-immune organs from each pig were taken for pathological analysis. For microscopic examination, the indicated tissues were fixed in 4% paraformaldehyde and then subjected to the preparation of pathological sections with a routing protocol. Briefly, the fixed tissues were flushed with running water overnight. After dehydration in a graded ethanol series, the tissues were clarified with fresh xylene and then embedded into paraffin. Serial sections of 4 µm thickness were prepared, followed by Hematoxylin and Eosin (HE) staining. To further examine the protein levels of LDHA in different tissues after CSFV infection, IHC exploiting a rabbit anti-LDHA antibody (Abmart, P00338) was performed. The Dako REAL EnVision Detection Systems (DAKO, K500711-2) was used to display the staining. After counterstaining with hematoxylin for 3 min, sections were dehydrated and then subjected to microscopic imaging with a NIKON Eclipse Ci biological microscope (Japan) under a magnification of 100×. For each tissue, three visual fields from three sections were randomly selected for further analysis. The appreciable brown staining is considered a positive protein with LDHA.

### L-lactate measurement

L-lactate levels in cell supernatant were assessed by L-Lactate Assay Kit with WST-8 (Beyotime, S0208S). In brief, 50 µL of diluted samples and standard solutions were added into the sample wells of the 96-well plate, and the wells containing only BeyoLysis Buffer A for Metabolic Assay or Lactate Assay Buffer were set as blank control wells. Next, 50 µL of WST-8 color working solution was added to each well, mixed well, and incubated at 37°C for 30 min away from light. Then, the absorption was measured at 450 nm. The standard curve value of each well was subtracted from the value of the standard zero-concentration well. Establish the standard curve and calculate the concentration of L-lactate in diluted samples (A), in which the value of the sample wells should be subtracted from the value of the blank control wells. The real L-lactate concentration in the samples is calculated as follows: C (mM) = A × n, where n is the dilution of the sample.

### DNA constructs, RNA interference, and transfection

The full-length porcine *LDHA* gene (GenBank, MG492003.1) was amplified by PCR, and the *LDHA* gene was cloned into p3×Flag-CMV vector by using *EcoRI* (Thermo Fisher Scientific, FD0274) and *KpnI* (Thermo Fisher Scientific, FD0524) to generate p3×Flag-LDHA. Primers used for the *LDHA* gene are listed in Table 1. p3×Flag-CMV is preserved in our laboratory.

**TABLE 1:** Primers for *LDHA* gene amplification

Gene	Sequence (5′−3′)
*LDHA*	F: CCGGAATTCATGGCAACTCTCAAGGATCAG
	R: CGGGGTACCTTAAAATTGCAGCTCTTTCTGG

siRNA against LDHA was synthesized by Sangon Biotech. Sequences of siRNAs are listed in Table 2. PK-15 and 3D4/2 cells were grown to 60% confluency in 12-well cell culture plates and transfected with plasmid or siRNA using Lipofectamine 3000 reagent (ThermoFisher, L300015) according to the manufacturer’s instructions. Briefly, 2 µg plasmid or 50 nM siRNA with or without 2 µL P3000 was diluted in 50 µL serum-free OptiMEM (ThermoFisher Scientific, company, 22600050), and 3 µL Lipofectamine 3000 was also diluted in 50 µL serum-free OptiMEM. The dilutions were mixed well and incubated at 25°C for 15 minutes. The mixture was then pipetted into 12-well cell culture plates and incubated for a further 24 h at 37°C.

**TABLE 2:** siRNA sequences of targeted genes used in this study

Gene	Sequence (5′−3′)
si*NC*	F: UUCUCCGAACGUGUCACGUTT
	R: ACGUGACACGUUCGGAGAATT
si*LDHA*	F: CCAAUCCAGUGGAUAUCUUTT
	R: AAGAUAUCCACUGGAUUGGTT

### Quantitative real-time RT-PCR (qPCR)

For targeted gene expression analysis, total RNA was prepared using Total RNA Kit I (Omega, R6834-02). Complementary DNA (cDNA) was synthesized using HiScript II Q RT SuperMix for qPCR (Vazyme, R223-01). Real-time qPCR was performed using ChamQ Universal SYBR qPCR Master Mix (Vazyme, Q711-02) and iQ5 iCycler Detection System (Bio-Rad, USA). Relative mRNA expression was assessed using the 2^−ΔΔCt^ method and normalized to the housekeeping gene β-actin. The primers used are shown in Table 3.

**TABLE 3:** Primer sequences of targeted genes used in this study

Gene	Sequence (5′−3′)
CSFV-*NS5B*	F: CCTGAGGACCAAACACATGTTG
	R: TGGTGGAAGTTGGTTGTGTCTG
*β-actin*	F: GGCACCACACCTTCTACAACGAG
	R: TCATCTTCTCACGGTTGGCTTTGG
*LDHA*	F: CTGTGTGGAGCGGAGTAAAT
	R: TGCTTTCCAGTGTTCCTTATCT

### Confocal immunofluorescence microscopy

Cells were grown in 35 mm petri dishes (Biosharp Life Science, BS-15-GJM) with a glass bottom. The cells were treated as described in the figure legends. When needed, mitochondria in live cells were stained with 100 mM Mito-Tracker Red CMXRos (Beyotime, C1049B) for 30 min at 37°C. Cells were washed with PBS and fixed with 4% paraformaldehyde (Sigma-Aldrich, P6148) for 30 min at room temperature; they were then permeabilized with 0.2% Triton X-100 (Sigma-Aldrich, T8787) for 10 min. The cells were blocked in PBS containing 5% bovine serum albumin (BSA; Beyotime, ST023) for 30 min. Next, the cells were stained with the indicated primary antibodies of rabbit and/or mouse in PBS at 37°C, followed by a 1 h incubation in PBS containing goat anti-mouse and anti-rabbit secondary antibodies conjugated to FITC and Cy3, respectively, at a dilution of 1:500. Wherever indicated, the nucleus was stained with DAPI (Beyotime, C1002). The fluorescence signals were visualized with a TCS SP2 confocal fluorescence microscope (Leica TCS SP8).

### Western blot

Cell monolayers were washed with PBS three times and incubated on ice with cell lysis buffer for Western and IP (Beyotime, P0013) containing a protease/phosphatase inhibitor cocktail (Beyotime, P1051) and 1 mM PMSF (Beyotime, ST506) for 30 min. The lysates were centrifuged at 14,500 g/min for 15 min at 4°C, and the protein quantification was performed by the BCA protein assay kit (Beyotime, P0012). Equal amounts of protein samples were denatured for 5 min in 5 × SDS PAGE loading buffer, and protein samples were resolved by 12.5% SDS-PAGE and electrotransferred to polyvinylidene fluoride (PVDF) membranes (Millipore, ISEQ00010), which were then blocked for 1 h at 37°C in PBS containing 5% (wt:vol) non-fat milk (Beyotime, P0216) in PBS with 0.05% (wt:vol) Tween 20 (Sigma-Aldrich, P2287). Next, the membranes were probed with primary antibodies at 4°C overnight and then exposed to the corresponding secondary antibodies conjugated to horseradish peroxidase at 37°C for 1 h. The protein bands were detected by the ECL Plus kit (Beyotime, P0018) and X-ray film exposure (Tanon, China).

### Co-immunoprecipitation (Co-IP)

Briefly, cells were lysed with a Western blot and IP lysis buffer (Beyotime, P0013) containing a protease/phosphatase inhibitor cocktail (Beyotime, P1050) in an ice bath for 20 min. Cells were scraped and then centrifuged at 14,000 g/min for 10 min at 4°C. A portion of the supernatant was mixed with 5× SDS PAGE loading buffer (Beyotime, P0015L) and boiled for 10 min to give a whole-cell extract (input). The remaining lysate was incubated overnight on a 4°C shaker with the specific antibody (IP assay). The following day, protein A + G agarose (Beyotime, P2055) was added and then incubated for 4 h at 4°C on a shaker. The lysis mixture was then centrifuged at 4°C for 2 min (2000 g/min), after which the supernatant was discarded and the precipitate was washed three times with pre-cooled PBS. Finally, 5 × SDS PAGE loading buffer was added to the precipitate, and the mixture was boiled for 10 min for western blot detection.

### Electron microscopy

To observe the effect of 6 mM L-lactate on mitophagy, CCCP-treated PK-15 and 3D4/2 cells were treated with 6 mM L-lactate or left untreated and grown in 10 cm dishes. Cells were washed twice with PBS and treated with 2.5% diluted in PBS glutaraldehyde, which was fixed at 4°C for 30 min. Cells were then collected in 1.5 mL Eppendorf tubes and further fixed overnight. Cell pellets were dehydrated with an acetone series and embedded in epoxy resin. Next, ultrathin sections were prepared and observed by a JEM-2010HR transmission electron microscope (TEM; JEOL).

### Molecular docking analysis

Sequences of proteins LDHA and ISG15 were acquired from the Uniprot database (https://www.uniprot.org/). Protein-protein docking was performed between LDHA and ISG15 to investigate the relationships by the AlphaFold3 (https://golgi.sandbox.google.com/). Pymol (version 2.4) was used to investigate protein-protein relationships and further visual analysis.

### Statistical analysis

Data were presented as mean ± standard deviation (SD), and one-way, two-way ANOVA, or unpaired student’s t-tests were performed using GraphPad Prism software (version 9.0.0). A *P* value of < 0.05 was considered statistically significant.

## RESULTS

### CSFV infection promotes LDHA expression and lactate production

**Fig 1:**
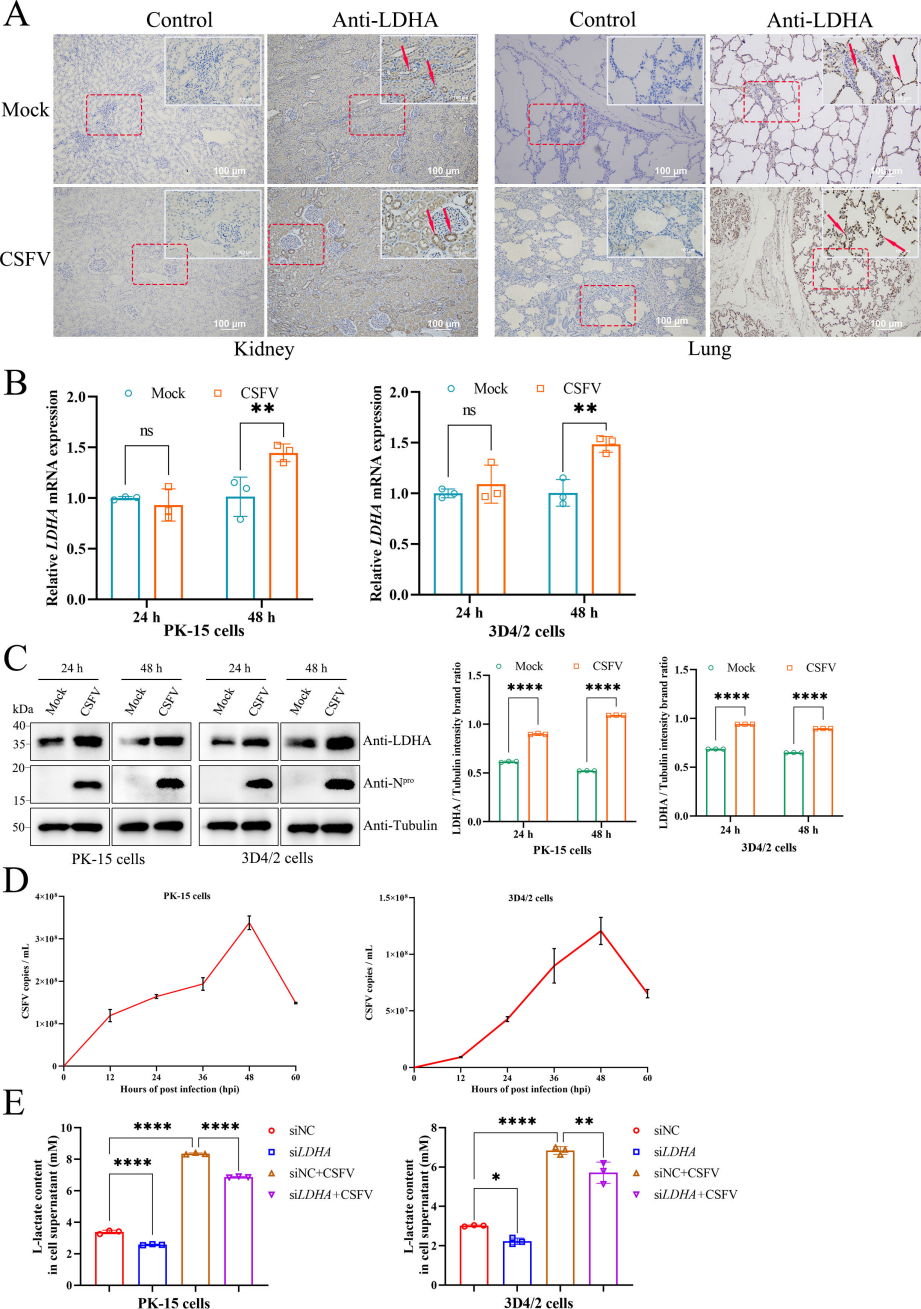
CSFV infection promotes LDHA expression. (**A**) Piglets were mock infected or CSFV infected for 7 days. IHC was utilized to verify LDHA protein levels in the lung and kidney. (**B and C**) PK-15 and 3D4/2 cells were infected with CSFV (MOI = 1.0) for 24 h and 48 h. The relative *LDHA* mRNA levels were assessed using qPCR as described in Materials and Methods. Western blot was performed to analyze the relative expression of LDHA, N^pro^, and Tubulin (loading control). The protein levels were quantified using Image J and visualized using the GraphPad Prism 9 software. Error bars indicate the mean (±SD) of 3 independent experiments. *P < 0.05; **P < 0.01; and ****P < 0.0001 (two-way ANOVA). (**D**) PK-15 and 3D4/2 cells were infected with CSFV for 0, 12, 24, 36, 48, and 60 h, respectively. CSFV copies were assessed using qPCR as described in Materials and Methods by detecting the CSFV *NS5B* gene. (E) PK-15 and 3D4/2 cells were transfected with siNC or si*LDHA*, followed by infection with CSFV or left uninfected. The L-lactate content in cell culture supernatant was measured by L-Lactate Assay Kit with WST-8. The data were visualized using the GraphPad Prism 9 software.

Our previous study has shown that CSFV infection disrupts glycolysis and results in lactate accumulation (13). To explore whether CSFV infection modulates LDHA expression, we selected six specific pathogen-free piglets, three of which served as the CSFV-infected group and the other three as the control group. Piglets were necropsied when they showed obvious clinical signs 7 days after infection with CSFV as described by Zhu et al. (21). LDHA protein levels were detected by IHC in the kidney and lung, as the two organs showing higher viral loads after acute infection with CSFV than other non-immune organs (21). The results showed that, compared with the control group, there were distinct dark brown labeled LDHA proteins in the kidney and lung on day 7 after infection with CSFV (Fig. 1A).

We then verified the role of CSFV infection on LDHA mRNA and protein levels, using qPCR and western blot, in PK-15 and 3D4/2 cells infected with CSFV for 24 h and 48 h or left uninfected as control. The results showed that CSFV infection for 24 h had no significant impact on *LDHA* mRNA levels, while it significantly increased *LDHA* mRNA levels in 48 h (Fig. 1B). Furthermore, LDHA protein expression was significantly increased after CSFV infection for 24 h and 48 h (Fig. 1C). We also examined the proliferation of CSFV in PK-15 and 3D4/2 cells at varying times after infection. It was found that CSFV copies increased continuously within 12–48 h after CSFV infection, peaking at 48 h and then decreased (Fig. 1D). Notably, the copy number of CSFV increased slowly at 12–24 h after infection, and the fastest at 24–48 h, which may be one of the reasons why *LDHA* mRNA levels did not increase at 24 h after CSFV infection but increased significantly at 48 h (Fig. 1D). In addition, we explored whether CSFV infection affects L-lactate content by modulating LDHA expression using the LDHA knockdown assay. The results showed that the L-lactate content in the supernatant of cells infected with CSFV after transfection with siNC was significantly elevated compared to the siNC-transfected group (Fig. 1E). Interestingly, the L-lactate content of cells infected with CSFV after knockdown of LDHA was significantly reduced compared to the CSFV-infected group after transfection with siNC, suggesting that CSFV infection is dependent on LDHA to induce L-lactate production (Fig. 1E). These data indicated that CSFV infection promotes LDHA expression *in vivo* and *in vitro* and induces lactate production *in vitro*.

### CSFV infection reduces LDHA ISGylation upregulating LDHA protein levels

**Fig 2:**
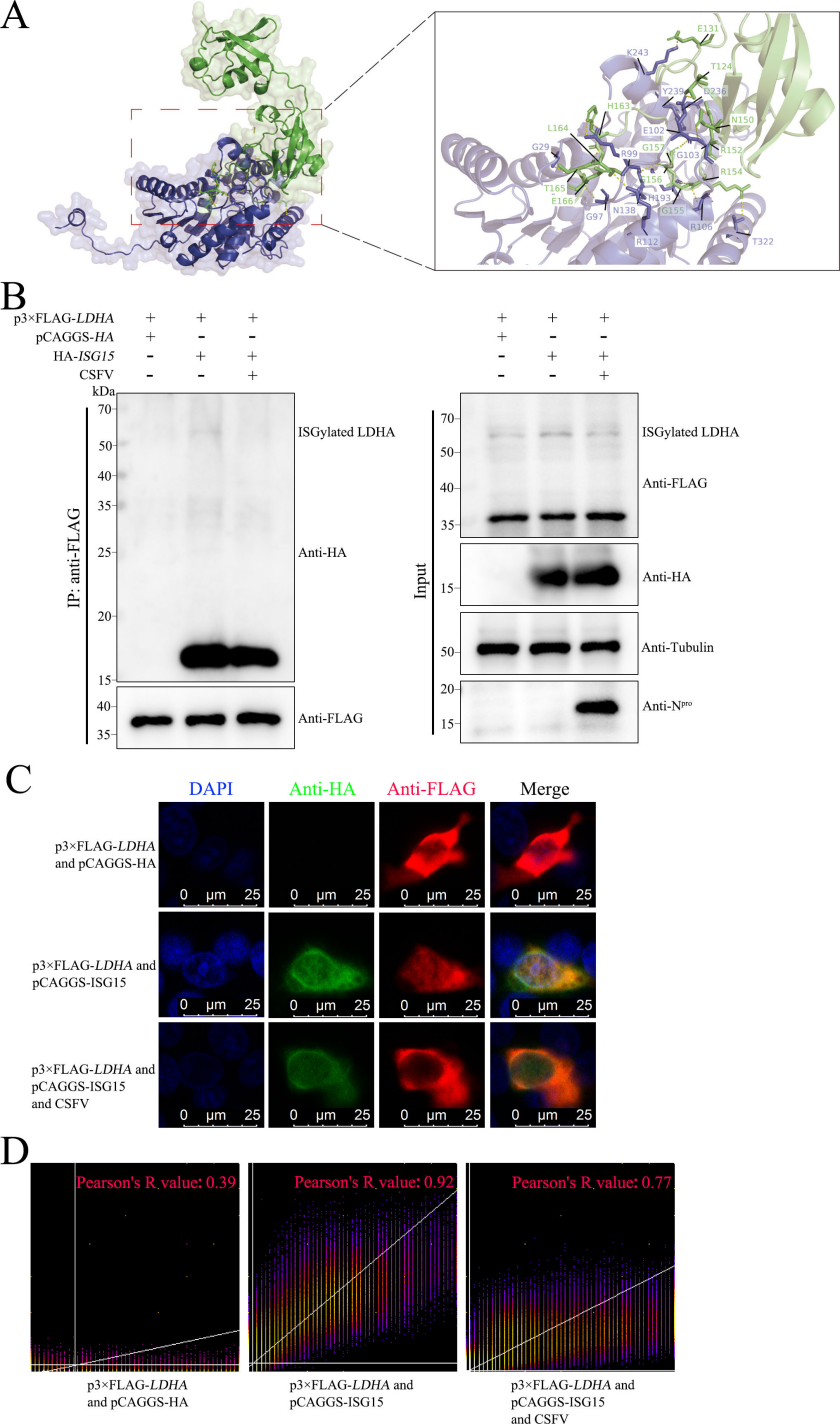
CSFV infection reduces LDHA ISGylation upregulating LDHA protein levels. (**A**) Surface diagram of docking model and their interfacing residues between LDHA and ISG15 protein (LDHA, blue; ISG15, green; hydrogen bond interaction, dotted line). (**B and C**) PK-15 cells were co-transfected with p3×FLAG-*LDHA* and pCAGGS-HA, p3×FLAG*-LDHA* and pCAGGS-ISG15, respectively, followed by infection with CSFV or not in the LDHA and ISG15 proteins co-overexpressed cells for 24 h. Co-IP and western blot were performed to validate the binding of LDHA and ISG15 using anti-HA antibody and anti-FLAG antibody. When observing the co-localization of LDHA with ISG15 protein, the cells were immunostained with DAPI (blue), rabbit anti-FLAG antibody (red), and mouse anti-HA antibody (green). The immunofluorescence signal was observed using a Leica SP2 confocal system. (**D**) The co-localization of LDHA and ISG15 was further analyzed using the Image J (Fiji) software.

Protein ISGylation is composed of the covalent binding of ISG15 and its protein target. We here performed protein-protein docking between LDHA and ISG15. Interestingly, LDHA and ISG15 formed hydrogen bonds through amino acid residue sites (such as G29-T165, G97-T165, R99-H163, R99-L164, E102-T124, G103-N150, N138-G156, H193-G155, R106-R154, R112-E166, K243-E131, D236-R152, T322-R154, and Y239-G157), revealing that proteins LDHA and ISG15 formed a stable protein docking model (Fig. 2A).

We proceeded to verify the interaction between LDHA and ISG15 and the ISGylation of LDHA using Co-IP and laser confocal assays. It was shown that HA-ISG15 protein bands were successfully detected in immunoprecipitated complexes co-overexpressing LDHA and ISG15 when immunoprecipitated with an anti-FLAG monoclonal antibody, which was reduced by CSFV infection (Fig. 2B). Notably, we observed distinct protein bands in the protein marker 50–70 kDa in both IP and input samples co-overexpressing LDHA and ISG15, which is regarded as the bands of LDHA ISGylation (Fig. 2B). Interestingly, CSFV infection decreased LDHA ISGylation levels and increased LDHA protein levels that did not undergo ISGylation (Fig. 2B). Furthermore, the green fluorescence labeled with ISG15 fused to yellow fluorescence with the red fluorescence labeled with LDHA (Fig. 2C). The co-localization analysis showed that the Pearson’s R value of the co-localization between LDHA and ISG15 reached 0.92, while the Pearson’s R value was 0.77 after CSFV infection, indicating that CSFV infection could attenuate the co-localization (Fig. 2D). Taken together, LDHA is able to perform ISGylation, whereas CSFV infection attenuates ISGylation of LDHA, upregulating LDHA protein levels.

### LDHA modulates mitophagy in PK-15 and 3D4/2 cells

**Fig 3:**
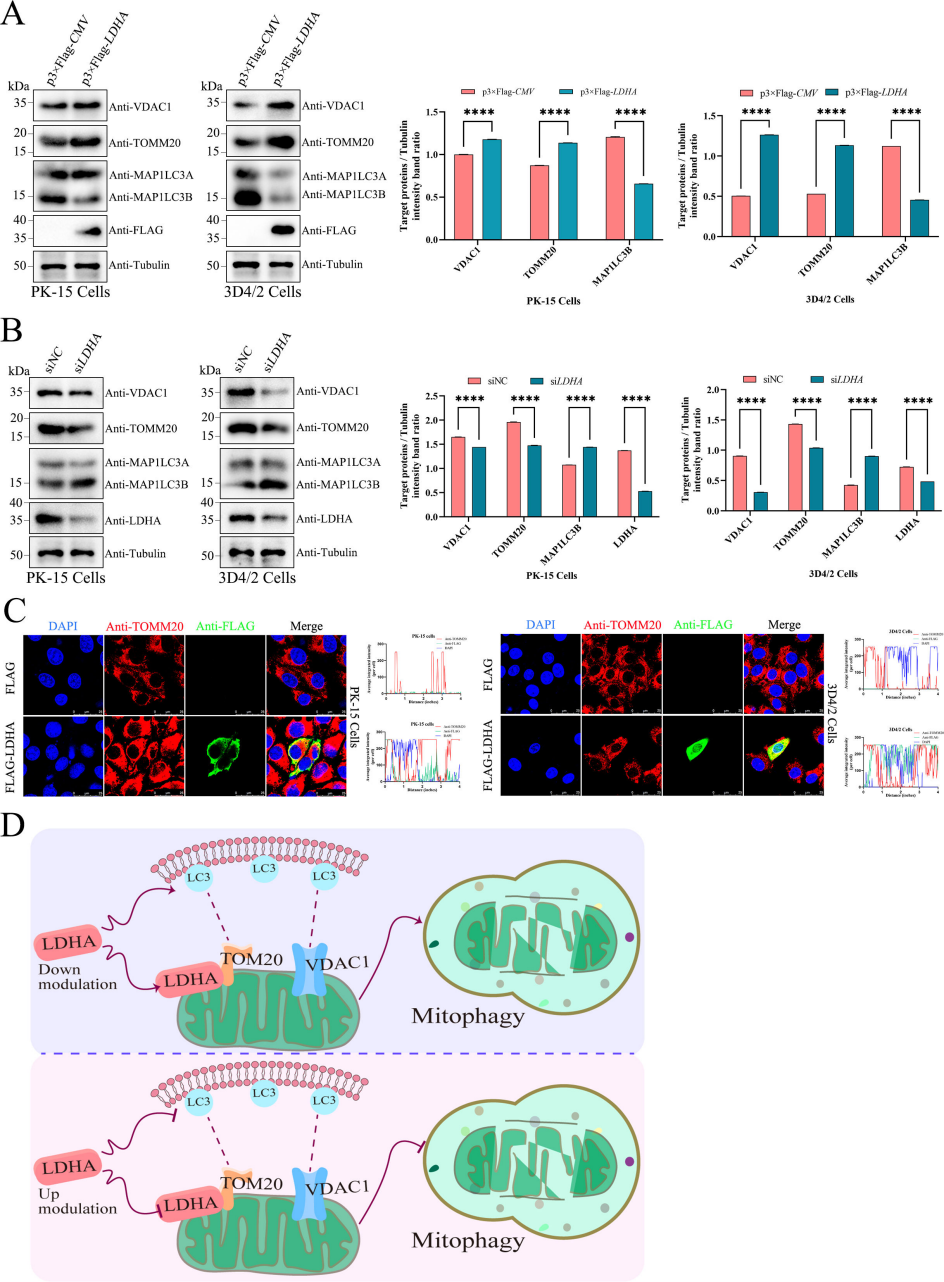
LDHA modulates mitophagy in PK-15 and 3D4/2 cells. (**A and B**) PK-15 and 3D4/2 cells were transfected with p3×Flag-CMV, p3×Flag-*LDHA*, siNC, or si*LDHA*, respectively. Western blot was performed to determine the relative expression of VDAC1, TOMM20, MAP1LC3A, MAP1LC3B, LDHA, FLAG, and Tubulin (loading control). The protein levels were quantified using Image J and visualized by the GraphPad Prism 9 software. (**C**) PK-15 and 3D4/2 cells were treated as in (Fig. 3A). Cells were immunostained with the DAPI (blue), anti-TOMM20 antibody (red), and anti-FLAG antibody (green). ImageJ (Fiji) software was used to calculate the fluorescence intensity of the line profile of the merged images and visualized by the GraphPad Prism 9 software. (**D**) Molecular pathway of LDHA-modulated mitophagy.

To investigate the potential role of LDHA in regulating mitophagy, we validated it by overexpressing and knocking down LDHA in PK-15 and 3D4/2 cells. The results demonstrated that LDHA overexpression significantly decreased autophagy marker protein MAP1LC3B levels and increased the levels of mitochondrial inner membrane proteins TOMM20 and VDAC1, compared with the empty vector transfection group (Fig. 3A); moreover, LDHA knockdown obviously upregulated the MAP1LC3B protein levels and downregulated the protein levels of TOMM20 and VDAC1, compared with the siNC transfection group (Fig. 3B). Laser confocal images showed that LDHA co-localized with TOMM20 in the cytoplasm, indicating that LDHA localizes in mitochondria (Fig. 3C). These data showed that LDHA modulates mitophagy in PK-15 and 3D4/2 cells.

### Lactate inhibits the PINK1-Parkin-mitophagy network

**Fig 4:**
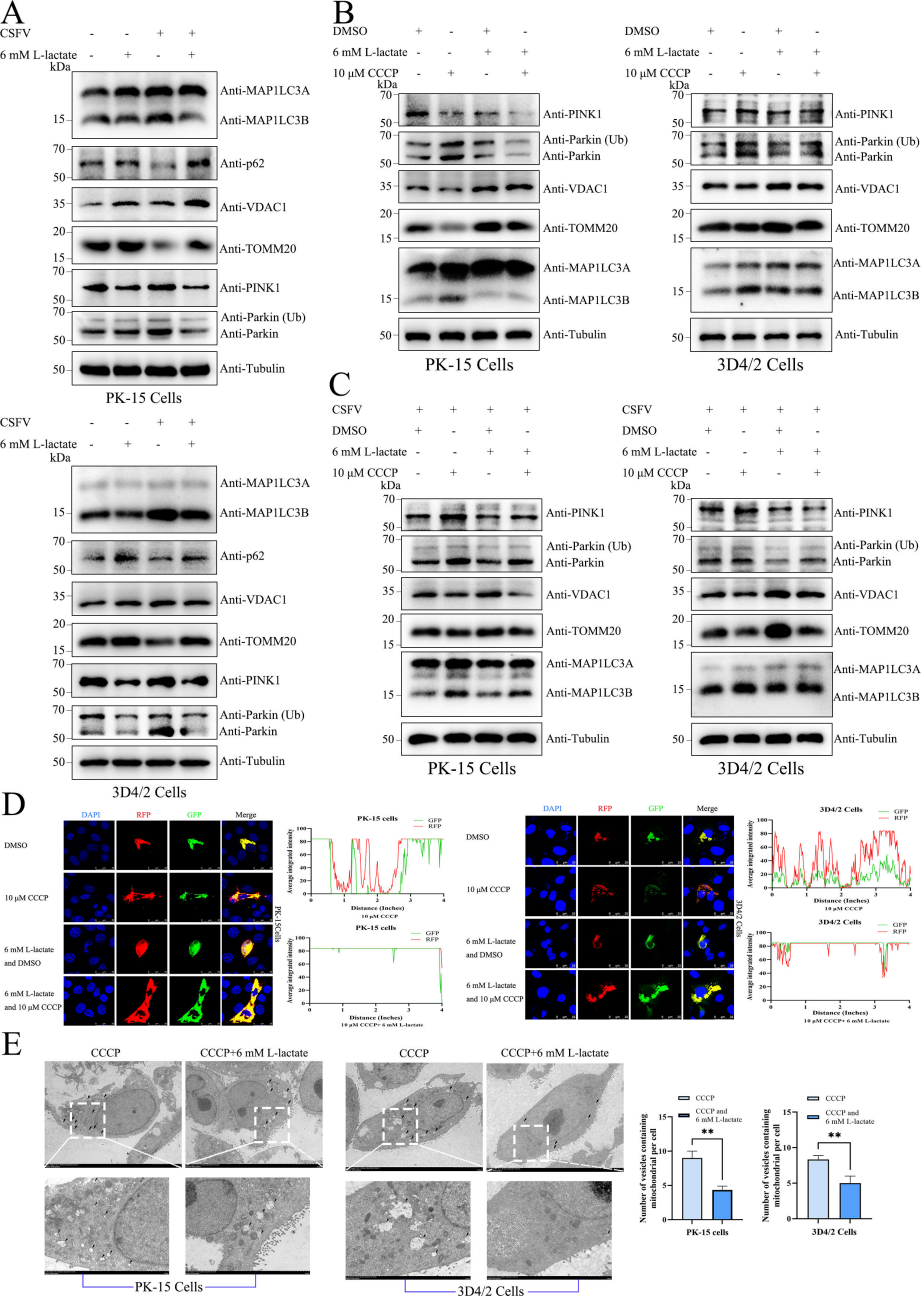
Lactate inhibits the PINK1-Parkin-mitophagy network. (**A**) PK-15 and 3D4/2 cells were mock infected or CSFV infected (2–4 h), followed by treatment with either 6 mM L-lactate or no treatment for 24 h. (**B**) PK-15 and 3D4/2 cells were treated with DMSO or CCCP for 4–6 h, followed by treatment with 6 mM L-lactate until harvest. (**C**) CSFV-infected PK-15 and 3D4/2 cells were treated as (**B**). Western blot was assessed to analyze the relative expression of MAP1LC3A, MAP1LC3B, p62, VDAC1, TOMM20, PINK1, Parkin, Ub-Parkin, and Tubulin (loading control). (**D**) PK-15 and 3D4/2 cells transiently expressing mito-mRFP-EGFP were treated as (**B**). Cells were immunostained with the DAPI (blue), RFP (red), and GFP (green). ImageJ software was used to calculate the fluorescence intensity of the line profile of the merged images, followed by visualization utilizing the GraphPad Prism 9 software. (**E**) PK-15 and 3D4/2 cells were treated with CCCP for 4–6 h, followed by treatment of 6 mM L-lactate or no treatment until harvest. The cells were then analyzed using a transmission electron microscope. Quantification analysis of the mitophagosome-like vesicles per cell image used GraphPad Prism 9 software (mean ± SD; *n* ≥ 3).

We further explored whether lactate modulates mitophagy. Our results demonstrated that lactate significantly decreased the levels of autophagy and antagonized CSFV-induced autophagy through the detection of autophagy marker proteins, MAP1LC3B and p62 (Fig. 4A). Moreover, we detected the mitochondrial inner membrane proteins TOMM20 and VDAC1. Our results demonstrated that lactate increased the quantity and quality of mitochondria and counteracted the decrease in mitochondrial number and mass induced by CSFV infection (Fig. 4A). Through the analysis of PINK1, Parkin, and Ub-Parkin protein levels, we found that lactate inhibited the PINK1-Parkin pathway and reversed CSFV-induced PINK1-Parkin activation (Fig. 4A).

To confirm the specificity of the inhibitory effect of lactate on mitophagy, we utilized CCCP, a specific agonist of PINK-Parkin-mediated mitophagy, for validation. Our results showed that lactate antagonized CCCP-induced mitophagy in both CSFV-infected and uninfected PK-15 and 3D4/2 cells (Fig. 4B and C). To evaluate whether lactate accumulation blocks the delivery of mitochondria to lysosomes, we transfected PK-15 and 3D4/2 cells with the mito-mRFP-EGFP plasmid, which displays mitophagy based on the differential stability of mRFP and GFP in lysosomes. The results revealed that cells co-treated with CCCP and 6 mM L-lactate had more yellow fluorescence than those treated with CCCP alone, indicating that fewer mitochondria were degraded by lysosomes (Fig. 4D).

Furthermore, the morphological changes of mitochondria were observed using TEM. Our TEM images showed no obvious changes in mitochondrial cristae in cells co-treated with CCCP and 6 mM L-lactate compared to those treated with CCCP alone (Fig. 4E). However, the number of mitochondria captured by membrane vesicles decreased in cells co-treated with CCCP and 6 mM L-lactate (Fig. 4E). Quantitative analysis also showed a significant reduction in the number of autophagosome-like structures in cells co-treated with CCCP and 6 mM L-lactate (Fig. 4E). Taken together, lactate specifically antagonizes the PINK1-Parkin-mitophagy network.

### LDHA-lactate axis regulates PINK1-Parkin-mitophagy network

**Fig 5:**
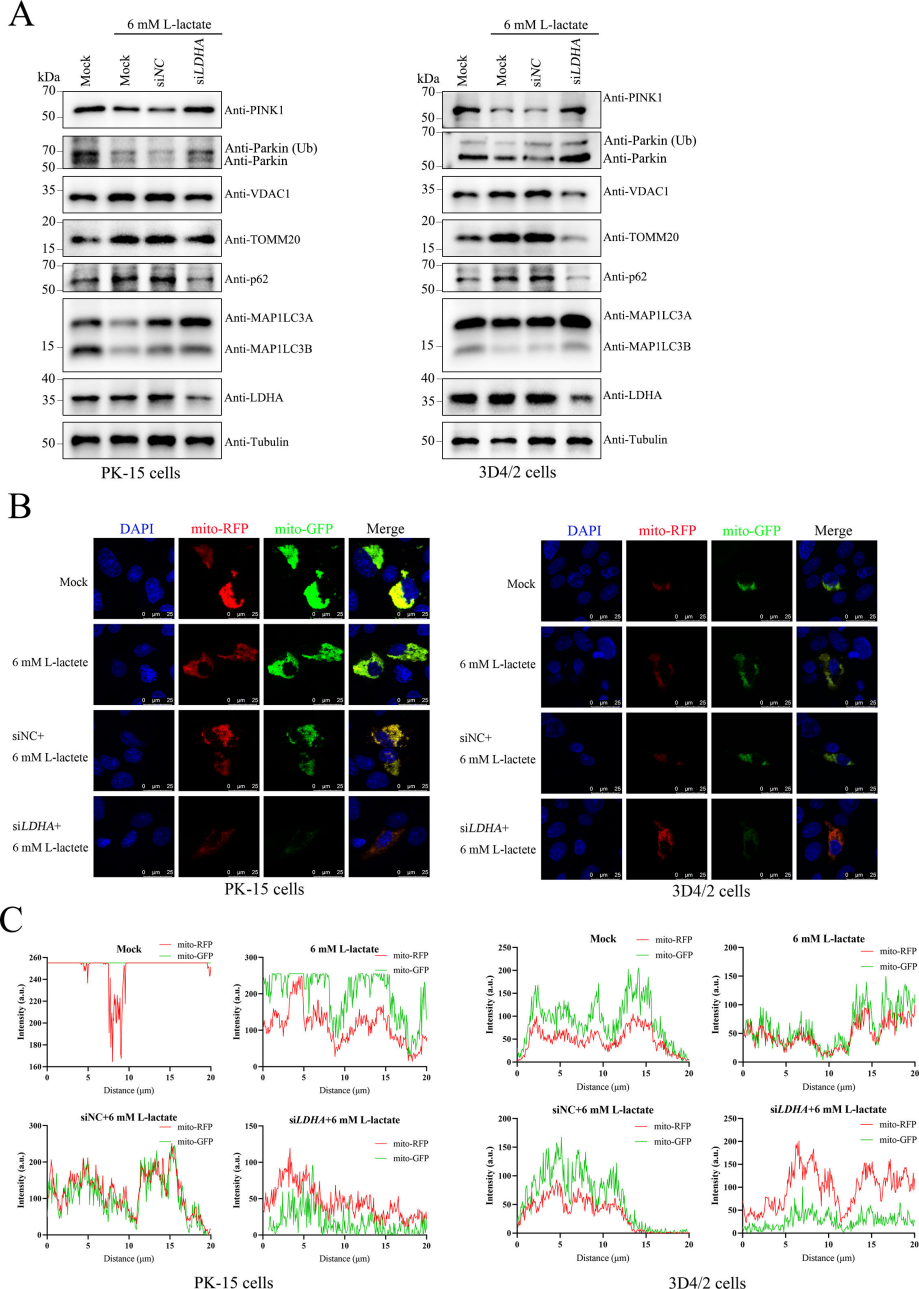
LDHA-lactate axis regulates PINK1-Parkin-mitophagy. (**A**) PK-15 and 3D4/2 cells were transfected with either siNC or si*LDHA*, followed by treatment with 6 mM L-lactate for 24 h. Western blot was performed to determine the relative expression of PINK1, Parkin, Ub-Parkin, VDAC1, TOMM20, p62, MAP1LC3A, MAP1LC3B, LDHA, and Tubulin (loading control). (**B**) PK-15 and 3D4/2 cells transiently expressing mito-mRFP-EGFP were treated as in (**A**). The immunofluorescence signals indicated the expression of mRFP and GFP proteins targeting mitochondria: yellow/green color, no mitophagy; red color, mitophagy, which were observed using a Leica SP2 confocal system. (**C**) Image J (Fiji) software was used to measure the fluorescence intensity quantitatively of the line profile of the colocalization image and visualized using the GraphPad Prism 9 software.

To further clarify the effect of the LDHA-lactate axis on the PINK1-Parkin-mitophagy network, PK-15 and 3D4/2 cells were transfected with siNC and si*LDHA*, respectively, or left untransfected as control, followed by treatment with 6 mM L-lactate or not for 24 h. We analyzed the protein levels of PINK1, Parkin, Ub-Parkin, TOMM20, VDAC1, p62, and MAP1LC3B using western blot. The results demonstrated that LDHA knockdown counteracted the inhibitory effect on the PINK1-Parkin-mitophagy network induced by lactate (Fig. 5A). Furthermore, PK-15 and 3D4/2 cells were transfected with a tandem-marked mRFP-EGFP plasmid encoding a mitochondria-targeting signal sequence, which was shown to be mitophagy based on the different stabilities of mRFP and GFP in lysosomes. Laser confocal images show that mitochondria in normal cells appear yellow because they carry both RFP and GFP fluorescence. By contrast, mitochondria in lysosomes only appear red due to the degradation of mitochondria by lysosomes (13, 22). As shown in Fig. 5B and C, PK-15 and 3D4/2 cells backfilled with lactate after the knockdown of LDHA showed more red fluorescence compared with control cells showing yellow fluorescence. The data indicated that the LDHA-lactate axis can modulate the PINK1-Parkin-mitophagy network.

### Lactate suppresses mitophagy upregulating the JAK1-STAT1 pathway

**Fig 6:**
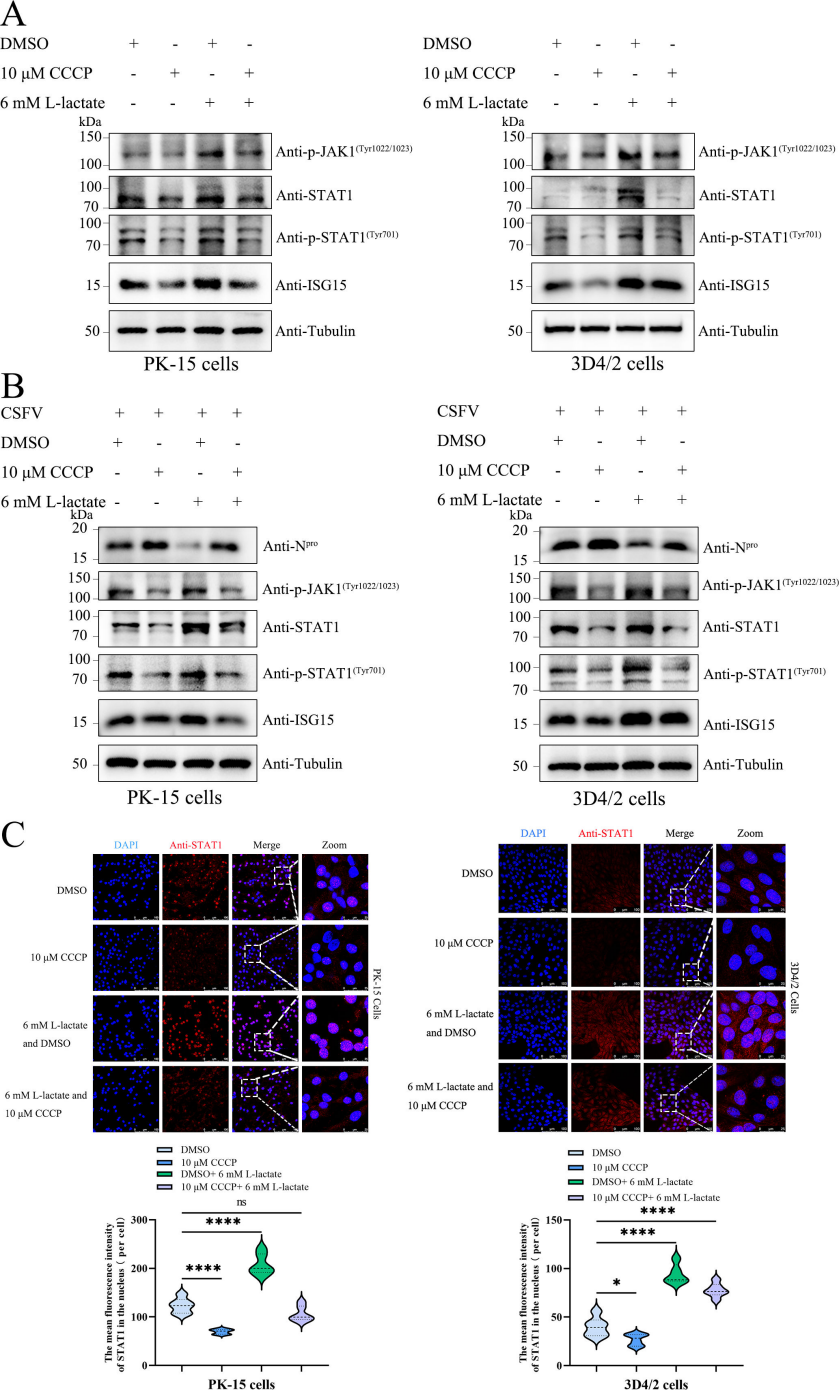
Lactate suppresses mitophagy upregulating the JAK1-STAT1 pathway. (**A**) PK-15 and 3D4/2 cells were treated with either DMSO or CCCP for 4–6 h, followed by treatment with 6 mM L-lactate or left untreated controls for 24 h. (**B**) PK-15 and 3D4/2 cells were treated with either DMSO or CCCP for 4–6 h, followed by infection with CSFV for 2–4 h and treatment with 6 mM L-lactate or left untreated controls for 24 h. Western blot was performed to analyze the relative expression of p-JAK1 (Tyr1022/1023), STAT1, p-STAT1 (Tyr701), N^pro^, ISG15, and Tubulin (loading control). (**C**) PK-15 and 3D4/2 cells were treated as in (**A**). Laser confocal detected the localization of STAT1 in cells. Cells were immunostained with the DAPI (blue) and the anti-STAT1 antibody (red). ImageJ software was used to calculate the mean fluorescence intensity of the merged images and visualized using the GraphPad Prism 9 software.

A study has indicated that there exists a regulation between mitophagy and the JAK-STAT pathway (23). Therefore, we further explored the relationship between lactate, the JAK-STAT pathway, and mitophagy. We treated cells with DMSO or CCCP and then treated them with 6 mM L-lactate or left untreated controls. The results revealed an obvious decrease in STAT1, ISG15, p-JAK1 (Tyr1022/1023), and p-STAT1 (Tyr701) levels in PK-15 and 3D4/2 cells treated with CCCP compared to controls (Fig. 6A and B). Notably, we found that lactate treatment counteracted the decrease in STAT1, ISG15, p-JAK1 (Tyr1022/1023), and p-STAT1 (Tyr701) levels induced by CCCP (Fig. 6A and B). Laser confocal images showed a significant decrease in STAT1 nuclear translocation in PK-15 and 3D4/2 cells treated with CCCP compared to controls (Fig. 6C). Furthermore, we found that exogenous lactate treatment counteracted the decrease in STAT1 nuclear translocation induced by CCCP in comparison to controls (Fig. 6C). Taken together, lactate activates the JAK1-STAT1 pathway through inhibiting CCCP-induced mitophagy.

### LDHA-lactate axis downregulates CSFV replication via mitophagy inhibition

**Fig 7:**
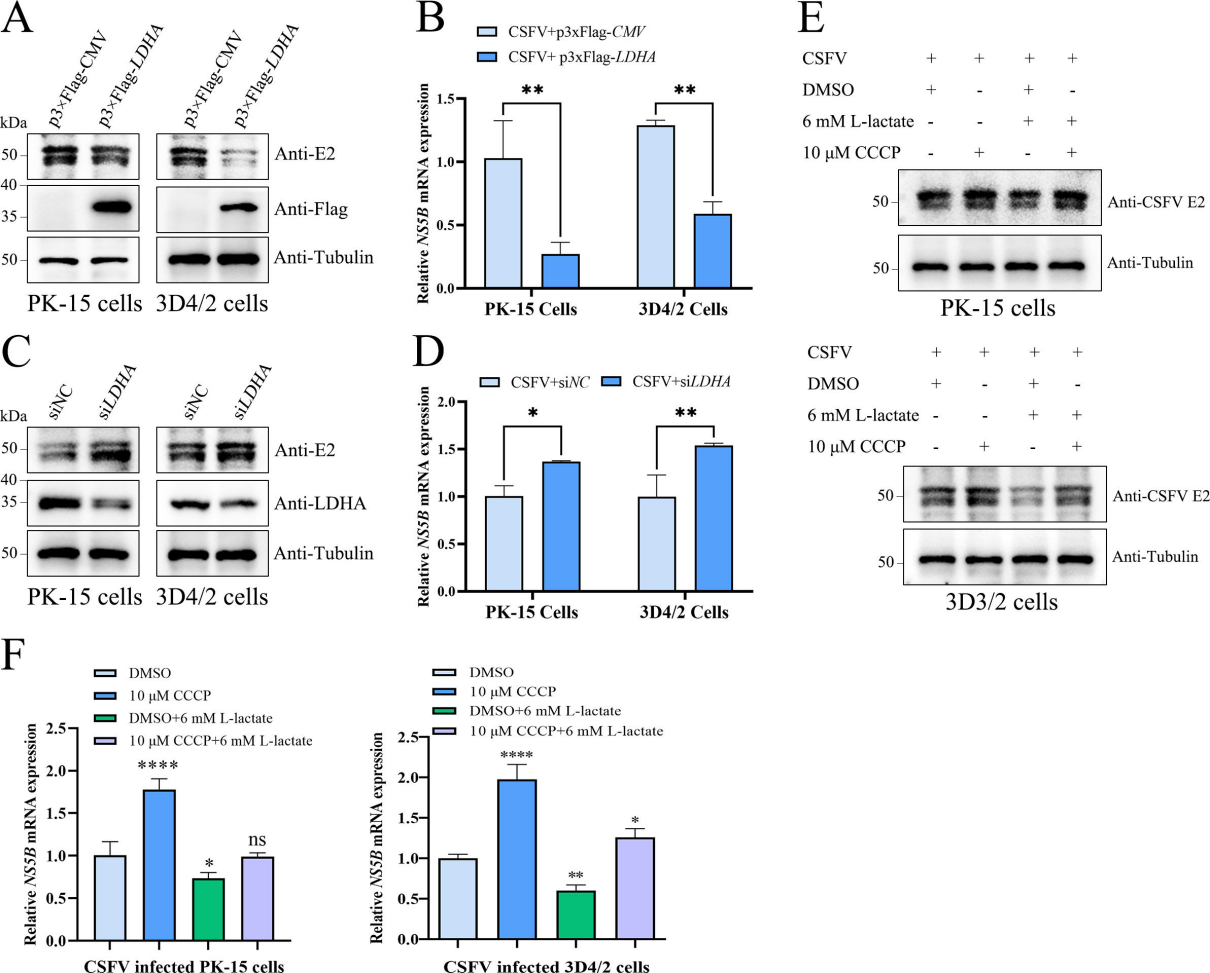
LDHA-lactate axis downregulates CSFV replication via mitophagy inhibition. (**A and B**) PK-15 and 3D4/2 cells were treated as Fig. 2A. (**C and D**) PK-15 and 3D4/2 cells were treated as Fig. 2B. (**E and F**) PK-15 and 3D4/2 cells were treated as Fig. 3C. Western blot was performed to analyze the relative expression of E2, FLAG, LDHA, and Tubulin (loading control). The *NS5B* mRNA expression was assessed using qPCR assay as described in Materials and Methods. Error bars indicate the mean (±SD) of 3 independent experiments. ns, *P* > 0.05; *P < 0.05; **P < 0.01 and ****P < 0.0001 (one-way/two-way ANOVA).

Finally, we investigated whether the LDHA-lactate axis affects CSFV replication through modulating mitophagy. As expected, our results demonstrated that LDHA overexpression and lactate treatment inhibited CSFV E2 protein expression and *NS5B* mRNA levels (Fig. 7A and B), whereas knockdown of LDHA promoted CSFV E2 protein expression and *NS5B* mRNA levels (Fig. 7C and D). Notably, lactate treatment counteracted the positive effect on CSFV E2 protein expression and *NS5B* mRNA levels induced by CCCP-induced mitophagy (Fig. 7E and F). These findings suggested that the LDHA-lactate axis downregulates CSFV replication via mitophagy inhibition.

## DISCUSSION

Viruses are strictly intracellular parasites that need to utilize the host’s energy and escape the attack of the host’s immune system (24). Lactate, as the end product of glycolytic metabolism, is no longer a metabolic waste product but serves as an important intracellular signaling molecule (25). The most striking function of lactate is to promote the growth of tumor cells by upregulating oncogenes and inhibiting CD8^+^ T-cell activation (1). Moreover, lactate boosts Toll-like receptor 4 (TLR4) activation and nuclear factor-κB (NF-κB)-dependent inflammatory gene expression via monocarboxylate transporters and myeloid differentiation protein-2 (MD-2) upregulation in human monocyte-derived macrophages and human U937 histiocytes (resident macrophages) (26). Significantly, in CD4^+^ T helper cells, lactate induces the switching of Th17 subsets to produce large amounts of the proinflammatory cytokine interleukin (IL)-17 (27). Lactate plays an important role in various immune cell differentiation and cytokine secretion. Correspondingly, lactate also plays a critical role in pathogen infection. A recent study showed that endogenous and exogenous lactate induces neutrophil extracellular formation of the network (NET), which contributes to bacterial and viral clearance (28). While NETs are crucial for pathogen clearance, they can also be involved in pathological conditions such as autoimmune diseases, where inappropriate NET formation contributes to tissue damage. It would be important to explore the mechanistic basis for how lactate influences NET formation and whether this effect varies across different cell types and infection models. Zhou et al. found that the hepatitis B virus (HBV) achieves immune escape by promoting glucose glycolysis to produce lactate and attenuating RLR-MAVS signaling (29). These highlight the dual role of lactate as both a product of host metabolism and a mediator of immune response during infection.

Lactate function depends on LDH, which catalyzes the conversion of pyruvate such that NADH and H^+^ are changed to lactate and NAD^+^ (30). LDHA modulates the maturation of human pluripotent stem cell-derived cardiomyocytes (hPSC-CMs) by inducing glycolytic metabolism (31). Researchers found that the overexpression of LDHA inhibits the AMP-activated protein kinase (AMPK) pathway and reduces mitochondrial reactive oxygen species (ROS) levels, thus elevating mitochondrial membrane potential (32). Previous studies in our laboratory found that CSFV promotes the accumulation of lactate and acts on viral replication through LDHB (13). However, the effect and mechanisms of LDHA on CSFV replication remain unclear. In this study, we found that CSFV infection promotes the expression of LDHA *in vivo* and *in vitro* (Fig. 1), which is related to the proliferation properties of CSFV (Fig. 1) and CSFV-reduced LDHA ISGylation (Fig. 2), indicating that LDHA may play an important role in CSFV replication. LDHA-mediated regulation of mitochondrial metabolism may also have broader implications for viral replication. Mitochondria are not only the powerhouse of the cell but also key regulators of the antiviral response. Viruses, including CSFV, may utilize functional and metabolic changes in mitochondria to promote their replication, and upregulation of LDHA by CSFV may be a strategy as well as a result of host cell-virus competition.

Mitochondria not only provide energy for life activities and metabolism but also are the primary site of aerobic respiration in cells, known as the “power shop” of the cells (33). Mitophagy is the main way to control the quality of mitochondria and plays an important role in innate immunity, cell growth, and viral infection (34). Interestingly, hepatitis C virus and CSFV induce mitophagy to attenuate the IFN response (13, 35). Considering CSFV promotes viral replication through mitophagy, we explored the role of the LDHA-lactate axis on mitophagy. We found here that LDHA not only inhibits mitophagy but also localizes in the mitochondria (Fig. 3), which may be involved in modulating mitochondria functions. Furthermore, our results showed that exogenous lactate significantly reduces the levels of CSFV-induced mitophagy and antagonizes PINK1-Parkin activation, but significantly increases the quantity and quality of mitochondria (Fig. 4). The data indicated that lactate inhibits the PINK1-Parkin-mitophagy network. To confirm the specificity of the negative effect of lactate on mitophagy, utilizing western blot, TEM, and pmito-mRFP-EGFP mitochondrial dual fluorescence plasmid, we found that CCCP treatment reverses the inhibition of mitophagy through lactate (Fig. 4). These results indicated lactate inhibits CSFV and CCCP-induced PINK1-Parkin-mitophagy activation. Importantly, we further demonstrated that the LDHA-lactate axis regulates PINK1-Parkin-mediated mitophagy (Fig. 5). The role of lactate in regulating mitophagy may have significant implications for cellular stress responses. Mitophagy is critical for the clearance of damaged mitochondria and the maintenance of mitochondrial integrity, especially during viral infections. By inhibiting mitophagy, lactate may prevent the turnover of damaged mitochondria, and damaged mitochondria may trigger an antiviral immune response. This effect suggests a possible novel interaction between viruses, metabolic regulation, and cellular immunity.

The JAK-STAT signaling pathway regulates plenty of viral replication, including HBV, oncolytic virus, SARS-CoV-2, porcine reproductive and respiratory syndrome virus, and CSFV (36–40). The study has shown that there is mutual regulation between mitophagy and JAK-STAT signaling pathways (23). However, whether and how the LDHA-lactate axis regulates the JAK-STAT signaling pathway remains unclear. Here, we further discovered that CCCP treatment can reverse the positive effect on JAK1 Tyr1022/1023 and STAT1 Tyr701 phosphorylation as well as ISG15 expression by lactate (Fig. 6A and B). Importantly, CCCP treatment is not only able to inhibit the nuclear translocation of STAT1 but also reverse the positive effect on STAT1 nuclear translocation by lactate (Fig. 6C). Our data suggested that lactate could activate the JAK-STAT pathway via inhibiting CCCP-induced mitophagy. However, the mechanisms by which lactate regulates the JAK-STAT-ISG15 network are still unclear. Furthermore, the connection between lactate, mitophagy, and the JAK-STAT pathway is intriguing because it suggests a metabolic checkpoint in the immune response that could be targeted for therapeutic intervention. By linking mitochondrial dynamics with immune signaling, lactate might be a key regulator of both innate and adaptive immunity during viral infections. Understanding how lactate modulates these pathways could open up new avenues for controlling viral replication, especially in chronic infections where immune evasion mechanisms are prominent.

Finally, we explored whether the LDHA-lactate axis modulates CSFV replication. As expected, the LDHA-lactate axis inhibits the CCCP-induced PINK1-Parkin-mitophagy network for restricting CSFV replication (Fig. 7).

This study sheds light on the LDHA-lactate axis, mitophagy, and CSFV replication. The findings discovered that CSFV infection increases LDHA expression. Furthermore, the LDHA-lactate axis inhibits PINK1-Parkin-induced mitophagy and activates the JAK1-STAT1-ISG15 network, suppressing CSFV replication. Our findings revealed for the first time the mechanism of the interplay between CSFV infection and the LDHA-lactate axis, providing a theoretical basis for the prevention and control of CSFV infection and may have broader implications for other viral pathogens.

## Data Availability

Data will be made available on request.
